# ‘A melting pot of cultures’ –challenges in social adaptation and interactions amongst international medical students

**DOI:** 10.1186/s12909-019-1514-1

**Published:** 2019-03-18

**Authors:** E. Byrne, R. Brugha, A. McGarvey

**Affiliations:** 1 Institute of Leadership, Royal College of Surgeons in Ireland, Ballymoss Road, Sandyford Industrial Est, Dublin, 18 Ireland; 2 Division of Population and Health Sciences, Royal College of Surgeons in Ireland, 123 St Stephen’s Green, Dublin, 2 Ireland; 3 Anatomy Department, Royal College of Surgeons in Ireland, 123 St Stephen’s Green, Dublin, 2 Ireland

**Keywords:** International education, Culture, Medical education, Acculturation, Internationalisation, Social adjustment

## Abstract

**Background:**

The internationalisation of higher level education and the profiles - nationalities, ethnicities and cultural identities - of students who migrate to undertake higher level education programmes in a different country are increasingly complex. This article explores the way in which cultural backgrounds impact the student’s experiences of an international medical school, and how these experiences have the potential to inform the development and design of student support services for those students who are not coping well with the transition.

**Methods:**

Thirty one first year students were interviewed by sixteen second year students who were trained and supervised by an experienced researcher. Three focus group discussions were also held.

**Results:**

While many international students had lived in more than one country and region and spoke several languages, most reported difficulties in forming intercultural friendships, especially interactions outside of the academic setting. Some of the challenges faced were similar to what has been reported in the literature, such as difficulties with language and loss of established friendship networks. Other challenges to emerge in this study were the complex interrelatedness of the daily life challenges facing international students regarding the forming and importance of intercultural relations, which is impacted by gender, the presence of alcohol, languages spoken (in addition to English, which was the language used for medical education), and the dominance of the regional grouping the student belongs to.

**Conclusion:**

The challenges of adaptation and intercultural relations are increasing in complexity and it is important for higher level institutions who enrol international students to understand the nature of the pressures these students experience, outside as well as within the academic environment, and to support them in managing these transitions.

**Electronic supplementary material:**

The online version of this article (10.1186/s12909-019-1514-1) contains supplementary material, which is available to authorized users.

## Background

Higher Education Institutions (HEIs) in Western countries host growing numbers of international students^1^. The number of international students has nearly doubled between 2000 and 2011 with almost 4.5 million students enrolled outside their country of citizenship [1]. Increasing numbers of students are opting to study at a university abroad [2] and an average of one in five students enrolled in higher level research programmes in 2011 was international [1].

Bennett et al. [3] in their review of literature on intercultural student relationships summarise the main aims and benefits of international education as: the potential to develop intercultural competency and prepare students to work in a global workforce; positive impact of intercultural interaction on international students, and; positive impact of intercultural interaction on host students in terms of awareness of different worldviews and developing an appreciation and respect for difference. Additionally, internationalisation of education is a well-documented financial asset for HEIs. Governments are increasingly recognising the opportunity and potential benefits of providing third level education to overseas students and the potential earnings from an essentially green and growing ‘industry’ [4].

International students generally have positive experiences, though sometimes they do not thrive in their new environments [5]. Jindal-Snapes’ Education and Life Transitions (ELT) model [6] addresses the relationship between a student’s academic transition and their daily life transition when integrating into a new environment; transitioning being “… an ongoing process that involves moving from one context and set of interpersonal relationships to another.” [7]. International students experience multiple and dynamic contexts in their new country, a new educational programme and a new educational system. Some of these experiences can act as stressors, with the impact of personal-emotional and social adjustment issues varying amongst international students, with some adjusting fairly easily and others experiencing considerable challenges [8]. Stressors can be related to either academic or daily life transitions. However, different experiences can happen in different aspects of the academic or everyday life. For example, doing well academically can overcome the negative experience of not being able to find your favourite food, and vice versa.

Smith and Khawaja [9] highlight acculturative stressors that if not addressed appropriately can lead to students not adjusting or integrating into the new environment in the way they wanted. In their review of acculturative stressors Smith and Khawaja [9] highlight that language and educational stressors are some of the main facilitators or barriers to acculturation across the body of acculturation literature they reviewed. Language barriers have implications in terms of academic and social adjustment. Socially, language barriers can inhibit international students making friends and interacting with host country nationals where their home language is not the same as theirs. Language anxiety can also increase academic stress. This stress contributes to, or magnifies, other stressors, such as a mismatch between students’ academic expectations and their initial experiences of university life and between expectations of performance and actual grades obtained, family/sponsor pressures, and different learning styles [8, 10, 11].

Additional stressors highlighted by Smith and Khawaja [9] include: *Sociocultural stressors* (from establishing a new social network; learning and adapting to new cultural norms and nature of friendships; and at times the transition from collectivist to individualistic cultures could have an additional impact); *Discrimination* (perceived or experienced, such as verbal insults, refusals of services or employment, and physical attacks, resulting in feelings of inferiority, depression, loneliness and homesickness), and; *Practical stressors* (such as financial problems, difficulties getting accommodation and negotiation of simple services such as transport). The limited research conducted during the early years of medical school [12–14] and cultural and linguistically diverse students in health professions education [15–17] confirms comparable results to the experiences of international students in general.

In transitioning having a good social network and supportive friends can help with the daily life and academic transitions. There is however, debate on the importance of whether this social network requires friendships with or without host students [18, 19]. Studies show that international students expect and desire contact with host nationals, but that in reality the level of contact tends to be relatively low [20]. Geographical origin, the region where a person is predominantly raised, is a significant predictor of adjustment, as is command of the English language [14, 19].

This article reports on the daily life transitions faced by international students in adjusting to their new environment and the implications this has for the international education of health professionals. Specific reference is made to possible strategies to facilitate early adaptation to the medical school and its setting and development of appropriate student supports.

A previous article [13] in *BMC Medical Education* by the same authors discusses the overall findings of both phases of the mixed- methods study that was conducted. This article reports on the same study, but focuses on a more in-depth analysis of the results from phase 1 of the research (interviews and Focus Group Discussions (FGDs)).

## Methods

**Fig. 1:**
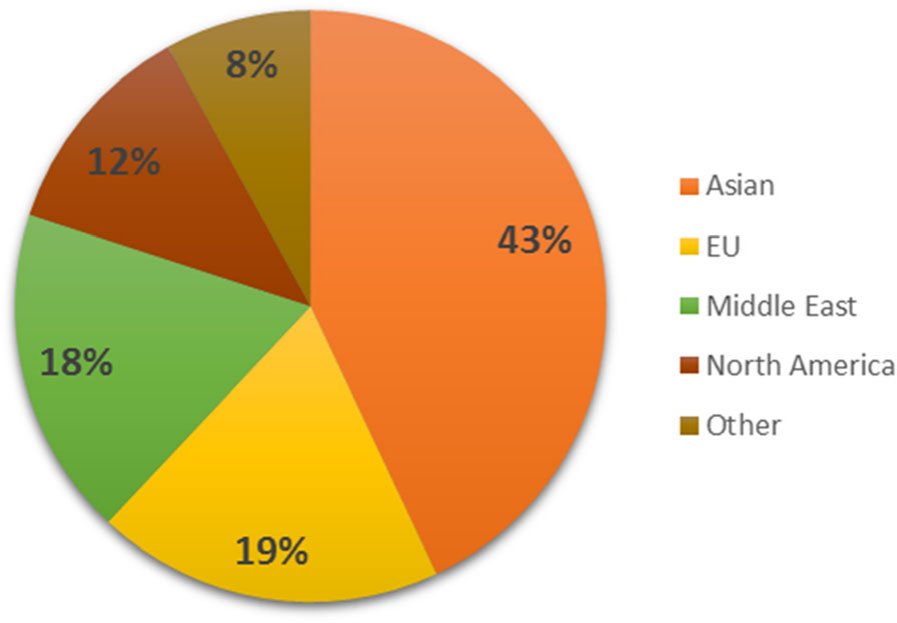
Representation of years 1 & 2 medical students based on region of origin for academic year 2014 / 2015 (adapted from [13])

The study was conducted in a standalone Health Sciences HEI situated in Ireland’s capital city, Dublin, which has an annual intake of around 360 students to its undergraduate medical programme. This HEI has historically hosted large numbers of international medical students. During the pre-clinical years (the first two or three years, depending on the student’s entry route), typically 80% of the students are from other non-EU countries, around 60% of whom are from Asia (mainly Malaysia) and the Middle East – see Fig. 1. This makes this HEI particularly suitable for exploring the transitioning and intercultural experiences of a rich mix of international students who are from quite diverse country and cultural backgrounds and form the great majority of the student body.

The study comprised a two phased mixed methods exploratory sequential study design [21, 22]. The study was approved by the HEI Research Ethics Committee (Reference number: REC566bb). The research team comprised the three authors (2 female and 1 male) who are all faculty members of the Faculty of Health Sciences and Medicine.

The first phase of the study was an exploratory qualitative phase as little research had been conducted in Ireland in this field. Sixteen Year 2 medical undergraduates were recruited and trained by one member of the research team -an experienced qualitative methods researcher - to conduct peer interviews with Year 1 students (peer interviewers). Peer or insider interviewing is discussed extensively in peer-reviewed journals [23] and recommended for primary health care clinicians in conducting qualitative research [24]. The method of peer interviewing in this study is published in more detail elsewhere [25]. Students were grouped according to the UN classification of geographic regions, though most students had multiple cultural backgrounds that are not necessarily represented by nationality. For example, an Irish student could have different nationality parents, lived/studied/worked in different countries as a child/adolescent, but have returned to Ireland to study. In this study this student would be classified as EU.

The volunteer peer interviewers were invited to attend two half-day training workshops. All peer interviewers (*n* = 16; see Table 1 for profile) were given a non-disclosure agreement to read and review in the first training workshop. All interviewers signed the non-disclosure agreement and returned it to the research team in the second workshop.

**Table 1:** Final profile of Year 2 student interviewers (*N* = 16)

	EU	North America	Other	Middle East	Asia	TOTAL
MEN	1	0	1	2	1	5
WOMEN	2	3	1	3	2	11
Total	3	3	2	5	3	16

All interviews (*n* = 31; see Table 2) were semi-structured, using open ended questions with a number of prompts to assist the interviewer in probing important issues. The interview theme sheet (Additional file 1) was developed based on the review of the literature and then piloted with the peer interviewers during their training. All interviews took place in the HEI, over a 2 week period in November 2012, 3 months after programme commencement. Students were paired by the researchers based on gender and self-declared cultural background/country of origin. All interviews were conducted in English. However, at times when additional clarity was requested on a line of inquiry the peer interviewers would also use a common shared language (in this case Arabic and Malay). All interviewees responded in English. All interviewees had a good command of English, which is a specific requirement for entry to the medical programme at this HEI - students must present a minimum overall average score of 6.5 with no individual section lower than 6.0. in the IELTS examination (academic stream). English language was the first language of the majority of interviewees and the majority of interviewees perceived their proficiency in English as excellent or adequate (see Table 3). While exact cultural matching was not possible in all cases, all interviews were matched for gender; and where possible by regional groupings. Matching for gender was based on the experience of the authors in other studies and is also supported in the literature where there is the tacit assumption that women are best interviewed by women [26]. In a parallel study on the peer interviewing for this study we found that the majority of interviewers felt that matching for gender was important [25].

**Table 2:** Profile of Year 1 student interviewees (*N* = 31)

	EU	North America	Middle East	Asia	Other	TOTAL
MEN	2	2	3^a^	2	0	9
WOMEN	2	6	5	6^a^	3	22
Total	4	8	8	8	3	31

^a^one interview not recorded/attended

**Table 3:** First language and perceived English language proficiency of students interviewed

Gender	1st language				Total	Perceived Proficiency in English			Total
	English	Arabic	Malay	Other		Excellent	Adequate	Poor	
Male	5	2	1		8	6	2		8
Female	12	5	2	2	21	14	6	1	21
TOTAL	17	7	3	2	29	20	8	1	29

A total of thirty-one students volunteered to be interviewed, one student did not show for the interview and one other interview was not recorded. Therefore a total of twenty-nine recorded interviews were obtained over the two-week period (referred to in quotes as JC 1 to JC29 where JC represents Junior Cycle – the 5 years of medical undergraduate training are divided into 3 cycles: Junior, Intermediate and Senior).

Interviewees signed a consent form after an information sheet on the research project had been sent to the interviewees via email before the interview. At the end of the interview the interviewees were asked to confirm their consent and were given a list of support service contact details in case they needed them. All interviewees were offered the opportunity to read their transcripts to check for accuracy and to ensure they were happy with the information they gave to be included as data for this study. Two interviewees availed of this facility and met with one of the researchers to read their transcripts. No changes were made to the transcripts as both were happy that the names they had referred to in the interview had been removed.

Three FGDs were then held with the same student cohort to explore further some of the issues raised in the interviews (see Additional file 2 for FGD guide). The FGD guide was shared with the peer interviewers to confirm the inclusion of these topics and whether other topics should be included. The themes that were explored further were stereotypes, and integration in relation to cultural practices, alcohol, language, teaching practices and assessment. The researchers were open to what possibly may not have been discussed in the interviews, explaining why alcohol, stereotypes, peer pressure were explored further. The FGDs were held after the students had transitioned from the junior cycle of the programme to the next year (intermediate cycle) of the programme and so additional questions on this transition were also included. The groups comprised 8–9 participants - one Middle Eastern (referred to in quotes as FGD ME), one Asian (was all Malaysian - referred to in quotes as FGD ASN), and one mixed (referred to in quotes as FGD MIX). The mixed group was held as there were insufficient number of volunteers to hold a separate EU, North American and other FGD. The authors of the article were the facilitators and rapporteurs for the FGD. As with the interviews participants signed consent forms and were offered the opportunity to read the transcript at a later date. No FGD participant availed of this opportunity.

All the data were inductively analysed thematically using NVivo 10, similar to that described by Denzin and Lincoln [27]. Initially coding was carried out independently on the first 10 interview transcripts by two members of the research team (referred to as ‘consistency checking’ by Newton Suter [28]). These codes were compared and common definitions of codes and a coding framework was developed. One researcher then recoded the 10 previously coded transcripts and the remaining transcripts using this framework following a similar process of coding and categorising as in Braun & Clarke [29] and commonly found in other qualitative studies [30] – these results are presented in the next section. This coding and categorising process included: familiarising ourselves with the data especially since we had not conducted the interviews, developing initial codes based on re-occurring patterns in the data collected, reducing the number of codes by combining codes into broader level themes, reviewing themes in relation to theories and literature, and agreeing on definitions and explanations of each theme. The FGDs were deductively analysed using the emergent topics from the interviews, though the authors were open to other themes emerging as FGD are analysed for content, but also for the process and group interaction [31]. The discussions, views and experiences of FGD participants were remarkably similar across the 3 FGDs.

The findings from the study were shared with the peer interviewers and interviewees after we held the FGDs and a previous version of this article was also shared with the Student Union and a number of the peer interviewers to validate the findings.

This study remains with some limitations. The interviews were conducted at one point in time though we are aware that the intercultural adjustment process is dynamic for international students [32]. Additionally, these interviews were conducted within 3 months of students commencing their first year as medical students in the host country and the FGDs were with students who had completed their first year. Some interviewees had already lived in Ireland for some time prior to commencing their studies. Though the respondents reflected the broader demographics of students in the HEI there were twenty-nine interviews and 3 FGDs conducted. The findings thus are the perspectives of these individual interviewees who volunteered to be part of the study. Whether participation is indicative of greater or lesser adjustment is unknown. Though the findings were shared with the interviewees they were not directly involved in the analysis and they may have coded the data in a different manner. However, the students were not trained in qualitative data analysis and this would therefore have involved additional training.

The second phase of this study measured and analysed the dimensions and issues highlighted through the interviews and FGDs across the entire 1st and 2nd year student body using an eighty-one item survey comprising items from the student adaptation to college questionnaire (SACQ) [33], the Social Integration Questionnaire (SIQ) [10] and questions to assess the representativeness of the qualitative findings. The findings from the second phase are reported in McGarvey et al. [13, 14]. As noted above this article focuses on a more detailed analysis of the qualitative results from phase 1 of the research (interviews and FGDs).

## Results

The categories that emerged are indicated in Table 4 and comprise negative and positive elements to the daily life transitions domain of Jindal-Snapes’ ELT model [6]. Overall, considering the positive and negative aspects of transitioning the main challenges faced by international students were around making friends and cross-cultural communication. Both the positive and negative aspects of transitioning in these themes are discussed together. Some of the other daily life challenges are briefly mentioned at the end of this section.

**Table 4:** Main categories, themes and codes from data in relation to positive (+) and negative (−) daily life transitions

Categories (Daily Life Transitions)	Themes	Codes
Making friends	Cross cultural relationships (+/−)	New social networks (−)
		Levels of friendships (+)
		Friendship attributes (+)
		Friendship facilitators (+)
		Commonalty of medicine (+)
	Meeting places (−)	Appropriateness of space (−)
		Alcohol (−)
	Personal space (−)	Greeting practices (−)
		Gender (−)
		Religion (−)
	Maintenance of own culture (+/−)	Dress code (+/−)
		Acceptable mixing (+/−)
Cross-cultural communication	Proficiency in English Language (+)	English demographics (+)
		Perceptions of proficiency (−)
		Implications of proficiency (−)
	Social proficiency in communication (−)	Colloquiums (−)
		Accommodation of diversity (−)
		Number of languages (+)
Other Daily Life Transitions	Discrimination (−)	Examples of discrimination (−)
		Implications of discrimination (−)
	Social/Work balance (+)	Managing household chores (−)
		Facilitators in attaining balance (+)

### Making friends

Making friends was the predominant category that emerged in relation to transitioning and was discussed in terms of developing cross-cultural relationships, the suitability of social meeting places and personal space.

#### Cross cultural relationships

Making friends was seen by interviewees as one of the main factors that determined how well and how quickly they settled into living and studying in the new host country. Most international students interviewed had left behind friendships and social networks built up over many years and the task of making new friends was daunting.

*That was something, I’d formed a lot of friends that are a big part of who I am now, eh so I kinda miss that,… [JC7]*

For some, it was easier to make friends with other international students as there was a common bond with others who were transitioning into living and studying in a new country. They perceived that the need for more intense relationships was something host country students wouldn’t understand as they already had their established social networks, which they had not left behind.

*Just the friends you make here, they understand what you’re going through. A lot of them are foreign students. They’re also struggling, don’t know anyone or know what’s going on at all. So the friendships, the intimacy or closeness is just so much stronger than I would ever have experienced before. …. The Irish, they just think you’re crazy. You want to plan or hang out and they have family and school friends, their whole life, they’re like what’s the big deal? They have a full life and your life is so empty. [JC8]*

The desire to make friends with host students was also articulated by some interviewees.

*I’d love to be friends with people from everywhere including Ireland because I’m here and it would be so great to have people who actually know Dublin and they’d be like, this is what we do, these are the suburbs and to get out there. There’s only so much you can try. I do try putting myself out there. [JC8]*

A clear distinction was made between different levels or types of friends (FGD Mix and FGD ME). One focus group participant (FGD ME) distinguished between class mates, friends and best friends. This indicated a clear difference between people you spoke to, people within the HEI that you would have coffee or lunch with, and then the close friends you would rely on if you were in trouble, or with whom you would socialise outside the HEI. International students interviewed also recognised that making friends required a lot of effort and brought challenges. Most felt that after making a certain number of close friends they did not try as much to expand their friendship base.

*I think it’s difficult in terms of language. When I first came I felt inferior, now I couldn’t be bothered any more. I think people don’t understand what you say kind of thing. You try your best to integrate yourself, but in the end you just feel like it’s too much effort you have to put in. You have Malaysian friends who you are comfortable with and then you just drift off. You just don’t put in that much effort. [FGD 3 ASN]*

Generally, international students interviewed reported little difficulty making friends with other international students, so in principle they perceived they did not find cross cultural friendships difficult to form. They attributed this to friendships being based on being comfortable with a person; understanding one another in terms of language, social norms and practices; having the same values; and sharing interests.

*I think it’s your hobbies, it’s what you do on the weekend that determines your friends. That’s for everyone, for Kuwaitis, Arabs, Norwegians, Malaysians, everyone. It’s the people you do hobbies with, you tend to stick around. [FGD ME]*

Sharing accommodation with people from other cultures was seen as a facilitator for intercultural interactions. In practice though, sharing accommodation also brought its challenges. An example given of such a challenge was when a Muslim student lent cooking utensils to her non-Muslim room mate who used them to cook non-halal products (FGD ASN). Additionally, several participants in one of the FGDs perceived that many students were not motivated to make friends outside their own culture or small core group [FGD Mix]. From the interviews there was a combination of interviewees who claimed they mixed very well across nationalities [JC1; JC10], whilst others reported that they tended to stick to certain groups [JC10]. Generally, mixing across nationalities and cultures occurred at a social level in the HEI, but was less common in close friendships or at events and activities outside/off campus.

*… there are different levels of friendships in RCSI, at least in our class. Take XXXX for example, I would consider her a friend, maybe she’s not in my close knit group, I wouldn’t text her, but if we sat on the bus together we would have a full conversation, how you going, how’s life, I haven’t seen you in so long and I think that’s a good thing. [FGD Mix]*

However, socialising within one’s own nationality or cultural background was not without some concerns. A few interviewees expected that if they did not dress or socialise in accordance with the expectations of their own cultural community, fellow students from their cultural background would talk negatively about them. A student from a Middle Eastern country that is more westernised said she ‘*felt serious pressure’* from others from her region to conform to what was perceived to be ‘her’ culture in relation to how she dressed, activities she engaged in, and with whom she socialised. The interviewee noted that this was entirely unexpected and negatively impacted on what the student felt should be a European experience [JC13]. Other participants in the FGDs also felt that there would be negativity from their peers, i.e. those from their own cultural background, if they did not conform to what was perceived as acceptable in relation to dress and with whom and where they socialised. The perceived negative judgements led one student to socialise with students outside her nationality, for another it made her adopt more conservative social behaviours.

*Coming here, my mum came with me a couple of times and she’s like, “Why didn’t I see you going to lunch with your guy friends?” I was like, ‘No mum this doesn’t happen here’. She was like “Why? In XXX [At home] you go …. Here you don’t”. I was like “No, because the next day the Arabs will sit and talk about me, the only girl with all the guys. She is so proud and so strong to go out with guys, what is she doing? ” I’ll be the ex-girl from the Arabs. I respect the Arab girls, that’s why I don’t interact well with the guys, whereas I’m perfectly ok with it. So I think they have influenced me and brought me back into my shell a bit which is strange but I’m admitting it. [FGD ME]*

Interviewees commented that they felt comfortable wearing the clothing of their choice or culture and were also comfortable with what other students wore. In many cases interviewees were relieved at the clothing worn by people in Ireland, which was generally seen as not ‘revealing’ (i.e. modest), even if it was the weather that accounted for this.

*I think people over here are free to practice whatever cultural traditions they have. If a Muslim girl has a hijab on that’s ok. It is accepted. [JC10]*

Interestingly, in the focus group discussions many of the participants said they were attracted to the HEI as they would not feel out of place attending class wearing their customary clothing, especially the *hijab*, because many of their fellow students would be dressed in this way.

Another factor that influenced friendships was the fact that the HEI is a stand-alone college of health sciences. As all students were medical students and mostly international they shared common interests and goals, therefore students could easily find a common starting point in conversations. Another feature that affected cross cultural mixing was the number of students from the same country studying at the HEI. Figure 1 shows that the largest regional groupings of students in this HEI were from Asia (mainly Malaysia), Middle East, Europe (mainly Irish) and North American (from the USA and Canada). Interviewees from under represented countries and those outside these dominant regions noted that they needed to reach out to mix and form friendships with students from different cultures. Lacking co-nationals, they generally had more intercultural interactions and friendships. Breaking into and forming friendships within these large geographical groups of people, such as students from the Middle East, was viewed by outsiders as difficult.

*The other thing I noticed is that there are a few big groups. I’m actually from Hong Kong so I kind of stick with the Asian groups and the others, … There are a few big groups so if you want to go from one big group to another and communicate with them it’s kind of intimidating. [FGD Mix]*

#### Meeting places

One of the main issues inhibiting intercultural interactions was the inappropriateness – from the perspective of many of the international students interviewed – of some social meeting places outside the HEI. One of the most popular meeting places in Ireland is the ‘pub’ (public house). In the Irish context the pub is mainly a venue to consume alcohol, although pubs are venues where food is also served. The difficulty with this meeting place for many international students interviewed was the presence of alcohol and the physical closeness and encroaching on personal space that they experienced in these settings. In the mixed nationality FGD there was an acceptance that going to the pub did not mean that you had to drink and individual FGD participants claimed not to feel any pressure to drink or any negative repercussions around not drinking. For those interviewed who drank there was the perception that socialising in a place where alcohol is served should not be a barrier to intercultural mixing. Most interviewees were not judgmental of others meeting in these places, though a number voiced their surprise at the frequency and volume of alcohol consumed by the host country students. However, for international students interviewed from Islamic backgrounds, going to the pub in itself may be considered by one’s network as a ‘bad thing’, whether the person wants to drink or not.

*The difficulty I have found in a multicultural environment like this is, for example, my relationship with my friends who are non-Muslim, that are not from Malaysia or the Middle East either. They live to go to the pub which is a really bad thing according to my religion because I am forbidden. It is prohibited in my religion to go to alcohol places. That is a negative thing that I need to tolerate. I’m not going to the pub to build a new relationship. Maybe have some nice coffee in the coffee dock and have a chit chat to build a new relationship with my other nationality friends. [JC28]*

In contrast some of the Irish participants in the FGD raised their discomfort on meeting outside the pub environment with the other students. For them having dinner in a friend’s house was intimidating as ‘*you have to go all in’* whereas in a pub you could chat with some people and then move on to another group if there was any discomfort [FGD MIX]. However, many interviewees were happy to experience the cultural differences and even to change some of their own practices whilst in an Irish environment.

Overall, alcohol was raised as a factor affecting intercultural relations outside of class or study time; and both drinkers and non-drinkers identified it as a barrier. Interviewees’ and FGD participants’ perspectives can be broadly categorised into 4 groups:

I. Drink heavily “*It’s part of my culture. I drink.”*
II. Drink moderately
III. Don’t drink *“I’m fine with drinking. I just don’t take part.”*
IV. Don’t attend events with drink *“If there’s alcohol there I can’t attend”*

Group I will attend alcoholic events as a preference and Group IV will not attend events where alcohol is present. Groups II and III are happy to attend events whether alcohol is present or not. Intercultural relations, outside study related activities, between groups I and IV are therefore unlikely.

#### Personal space

Another factor influencing intercultural interactions was personal space. Many of the interviewees were aware of differing practices different students had regarding hugging or shaking hands. Interviewees were aware that by not accepting a hand shake they might be viewed as discourteous or rude; and so for many they only shook hands when this was initiated by others. Others simply forgot at times that some of their friends would not be comfortable shaking their hands or would not be welcoming of a ‘birthday hug’ and would themselves feel uncomfortable with the stiff reaction received – ‘*either way it can be offensive’ [JC29]*. The experience from the perspective of a female international interviewee is illustrative and helps explain the need to look to one’s own social network for support in managing uncomfortable situations.

*For example, if I had an encounter with someone in my class, like I don’t necessarily shake hands with men because my religion doesn’t allow me to do so but some Irish fellows don’t know that and would be like, here, let me give you a big hug and I just freeze there. I can’t go to an Irish girl and be like, ‘Oh God, he gave me a hug’. She’d be like, ‘What the hell is wrong with you? He was being nice’. If I went to XXX I’d be like ‘Oh my God, he gave me a hug and I just stood there, I didn’t know what to do. I didn’t know how to react’ and she’d be like, ‘It’s ok. You can’t control that stuff’. In that sense she’d know where I was coming from. [FGD 2 ME]*

Underlying the cultural implications of personal space were gender and religious dimensions. Largely it was Muslim students who tended to socialise in gender specific groups – men with men and women with women [JC 10]. The issue of gender was also raised in the FGDs (FGD ME and FGD ASN) where the view was held by many of the Middle Eastern and Malaysian students that male and female friendships within the HEI were acceptable, but that this would not be acceptable in a social event outside the HEI.

*It’s not ok to hang out with a girl as a friend. You can’t just knock on her door and say, do you want to go for a coffee or I’m going to the park, do you want to head down? …. . [FGD 2 ME]*

Conversely, what was deemed appropriate to most interviewees from these cultures could be viewed as offensive to others. In one case a female interviewee was completely surprised that men and women did not sit next to one another and interpreted this as ‘ *Some of the men are in fact really rude’* and that these male students felt that they were ‘*better than you’* [JC25]. This misunderstanding of the others’ culture, especially around personal space and women wearing hijabs (headscarves), was also raised in the FGD.

*… some of the Irish guys they’d be very intimidated to approach me and just assume that since you have a head scarf on you are untouchable. They just go, ‘I don’t want to get involved in that. I don’t even want to say hello. I don’t want to offend her’. … We can be approached. … We have different boundaries, but it’s totally normal to walk up to a person in class and just have a normal conversation with them. [FGD 2 ME ]*

What was a concern to interviewees from the Middle East and Asia, who were predominantly Muslim, was the lack of understanding by host country (Irish) students of their culture*.*

*I find the problem here is they don’t really understand basic things in Islam like halal. … They don’t see why you cannot join a party even though you don’t drink. There are more things you have to consider. I can’t mix around. [JC30]*

### Cross-cultural communication

English was the first language of seventeen of the interviewees, 8 of whom had attended an international school prior to joining RCSI. In general, interviewees felt that they had sufficient English language skills to perform well academically – not surprising given that a minimum level of academic English is required for entry into the HEI. However, even with this level of English, second language English speakers, noted that they often had to work harder to attain their grades as many felt the need to translate the lectures to their home language and back again. This also meant that they felt unable to ask questions in the lectures as by the time they had translated the question they had to English the lecturer would have moved on to another point. One of the consequences of their ‘silence’ in terms of the perceptions of other students was that some first language English speakers could not understand how it was possible for international students to obtain good marks academically in exams through English, yet still not have the fluency or confidence to socially or engage in a class discussion, as evidenced by a participant in a FGD.

*With regard to the language barrier I find it hard, though with some people who seem like they can’t have a conversation with you, so how are they doing their ward tutorials? How are they doing their card signings, how are they reading their books and passing exams if they can’t have a conversation? So I don’t like to believe that they can’t speak English and want to believe that they don’t want to if that makes any sense. Clearly they’re succeeding in medical school so they must have rather proficient English. [FGD Mix]*

There was an overwhelming sense of embarrassment expressed (or the potential for this to occur) when international students interviewed made mistakes in English in front of others in lectures or practical classes. In many cases it was the self- perceived fluency in the language that caused the lack of confidence, rather than the actual ability.

*I think with me it’s more like when you feel inferior when you talk to people who you think have better English. And because I think, even in class, not many people, because sometimes the lecture is interactive, some are really confident. They are the minority. ... I can talk, just talk but my English and also my vocabulary, I can say my vocabulary is just bad. I don’t know much variety of English words .... [FGD ASN]*

The interviewees and FGD participants, however, distinguished between competency in English for academic purposes and competency in social situations [JC10]*.* This also contributed to a lack of comfort/ease in joining in conversations.

*The thing is about it, in a comfortable conversation you need to know how to express yourself and you feel comfortable with people. For me I’m not used to expressing myself talking in English. English is more for a presentation in class. I do talk in my secondary school a lot with my English teachers and stuff so I get the hang of English language. But the thing is I’m not used to being the crazy me talking in English! That might be an obstacle for me compared to when I speak Malay I know how to use the words, all the slang. You’re more comfortable with it. You can talk whatever you want in that language. It’s kind of like another obstacle for me. [FGD ASN]*

Interviewees felt that lecturers made an effort to be understood in terms of their language, but found that Irish students didn’t tend to adapt their language in conversations with international students. Furthermore, the English used outside the college was difficult to understand due to the different accents. Overall, many of the interviewees found it hard to understand the pace, accents, colloquialisms, ‘slagging’^2^ and jokes. Interestingly, other interviewees who were not speaking the dominant first languages of international students - Arabic or Malay - also felt intimidated to socialise in larger groups of international students.

*The thing is that when you walk into our lecture theatre Arabic and Malay are the dominant languages that are going on. It’s difficult for the Irish people to go into that situation where people are speaking in another language. It’s reciprocated both ways, but it would be hard for people whose friends all don’t have the same standard of English. [FGD Mix]*

For many interviewees and FGD participants the ability to speak in a number of languages enabled a more effective means to communicate a message than using a single language.

*… it’s different when you, for example, how you express yourself. I have a lot of Arab friends, not because they’re Arabs - it’s just that I find it easier to express myself in both languages instead of just one. Sometimes it’s difficult to express yourself fully in one language. [JC20]*

This is confirmed by another interviewee (JC2) who also noted that having command of another language, that is often your mother tongue, can help explain more fully the English meaning of words and this ability affects the people, and the way, you socialise with them.

In addition to the normal challenges of student life, in making friends and managing cross-cultural communications, there were specific instances or experiences, outside of the educational institution, that interviewees and FGD participants experienced as being discriminatory. These included attitudes of staff in restaurants; comments made or being shouted out on the street; occasional bad experiences of physical confrontation on the main city centre streets at night, which is a time when some passers-by have consumed excessive alcohol; and in some cases snowballs being thrown at them. Most interviewees did not experience major problems in balancing work and life and did not feel homesick or lonely. However, most had lived away from home on previous occasions, prior to entering medical school. Some interviewees (including Irish interviewees) had initially experienced difficulty in striking a balance between study, social life and the normal tasks of daily living such as household chores.

## Discussion

In a world of increasing internationalisation, it is clear that cultural backgrounds impact students’ experience of an international medical school. The following discussion outlines the results of this study in the context of existing literature. These findings can inform the development and design of early student orientation and adaptation programmes; and support services to enhance the student experience and provide support to those students who are not coping well with the transition. We also recommend that the information recorded about students’ needs to be adapted during the admission process to be more in keeping with this era of globalisation and education. Additionally this study highlights the importance of hearing the student voice and lived experiences in informing the type of additional supports that may be needed [15].

Much of the findings reflect how the students negotiate their daily life transitions and accommodate their own cultural identity in the context of managing intercultural relationships, and how they negotiate an understanding of others' cultural identity. This is apparent in the two main themes identified in this study – ‘Making Friends’ and ‘Cross cultural communication’. In the former students use the commonality of the medical college and shared international experience, and in the latter the students used a combination of languages to enhance meaning making, message transmission and negotiating new relationships. The ELT model highlights the identity negotiation and adjustments in their daily life transitions, but also indicates how the boundaries, between the positive and negative aspects of transitioning are negotiated.

A number of the stressors or challenges facing the interviewees and FGD participants in this study are similar to those reported in the literature. For example, cross-national friendships often develop between those who share similar experiences and are open to learning from other cultures [18, 34]; international students have difficulties with establishing friendships with host students due to language difficulties [10, 16, 18, 35]; English language skills to gain entry into a university cannot be equated with social competence in the same language [16], and; host students having already established friendships [34, 36].

There were a number of notable findings that to date have not been well highlighted in the literature on the negotiations within daily life transitions, namely negotiating social venues with/without alcohol present; intercultural communication through multilingual ability, and; presence or lack of a large stable ethnic community.

Other studies have reported the socialisation constraints experienced by students from some cultural backgrounds, not only among those from a Muslim background, in relation to how and where alcohol is served, consumed or is simply available [5, 37]. This study deepens knowledge about international students’ experiences, outlining four categories of alcohol consumption as identified above (Drink heavily; Drink moderately; Don’t drink; Don’t attend events with drink). These range from those who drink heavily to those who don’t drink and cannot mix in social circles where alcohol is being consumed. This categorisation may be useful for faculty and administrative staff when planning social events for international students. In addition our study highlights that it was not only the consumption of alcohol that was experienced by some interviewees as a constraint and deterrent to socialising in this country setting, but also the close contact between men and women in these social settings. The situation was aggravated by the often undesirable or anti-social behaviour of some people, mainly from the host country, when they had been drinking alcohol. Overall, it is unlikely that any social event will be attractive to all people, though in one of the FGD many of the interviewees felt that *‘sport was the universal language’,* as was food, and might be the common point from which to design an international activity. For students who are having difficulty adjusting, promoting and enabling them to participate in such sporting or food related activities are important.

An area of interest around language in this particular HEI, and increasingly common across the education sector due to the more globalised world we are living in, was the multilingual ability of the interviewees and FGD participants. It is quite common for students to have a number of different languages in which they communicate with one another. This assisted many in developing intercultural relationships, though not necessarily with the host students. Socially, given the national and regional composition of the student body within this HEI (see Fig. 1), English is not the dominant social language spoken, even if it is the language of tuition and learning. Communicative competence development thus takes place in the socialisation process [38] and this can pose challenges associated with code-switching and load in relation to successful informal and formal interactions [17]. For some English language students this inhibited their attempts to mix or interact with the dominant groups, where Arabic and Malay were more often the languages of socialisation (Table 3). These challenges will continue for the students as they graduate and are practicing as doctors around the world. Indeed highlighting the use of language as part of cultural competence with the students as a whole would be helpful in making them more conscious of their use of language. This is especially the case given that English is the universal language of medicine. This particular HEI could harness the collective transitional experiences and cultural capital of the linguistically diverse students to inform communicative practices in the increasing culturally and linguistically diverse hospital settings worldwide [17].

Interestingly those interviewed not from a large cultural or national group body present in the college tended to have more mixed intercultural interactions, both with the host country and with other minority groups. This parallels Volet and Ang [19] identification of ‘left overs’ in class being a catalyst for intercultural interactions in a university context. It also echoes Rienties et al’s finding: “… when the international students came from smaller nationality groups, they were seen to integrate well with host students or international students from other countries, as the need to develop links outside one’s culture probably was stronger for these students.” ([39], p501). We found that a large stable ethnic community can also have disadvantages in terms of perceived ‘chit-chatting’ within the community about the behaviour of other students from that community, e.g. about their dress or with whom they are socialising; as well as placing pressure on them to conform to what is considered appropriate for that community. For some students in these large regional groups this alienated them from socialising within their nationality groups or caused them to adapt their behaviour and be more conformist. Our findings highlight that what is important to the interviewees is having a social network in which they are comfortable to be themselves and practice their culture in a way they feel is appropriate for them whether this network comprises the same or a cross section of different nationalities. Alienating students from their own cultural group can have implications for those students returning to their home countries post–graduation and who will need to be able to be comfortable in that society.

Gender plays a large role in the forming of cross-gender and cross-cultural friendships. In this study, the role of gender was influenced by the prominent number of Muslim participants who were from the Middle East and Malaysia, which has implications for the level of close friendships that can be made between male and female students even within the same nationality grouping. The gender dimensions to intercultural interactions in particular relating to personal space and dress has not been highlighted in other studies, though issues around personal space in general have been [16]. In the interviews it was mentioned that the women would not be able to go to the gym^3^ when men were present because they would have been spoken about. Likewise in the FGD not conforming to perceived cultural norms in relation to dress or socialising with the opposite sex would lead to stories about them, questioning their fidelity to their nationality or culture. While there is a clear association between gender practice and religion, mainly within Islam but also within conservative Christianity, there was also a distinction and differences across religion, culture and traditional practices, as articulated by participants in the FGDs. The degree of acceptable socialising of men and women varied amongst the student group and depended on religion, country of origin, how conservative one’s upbringing was, the venue type, and how comfortable the individual was in mixing with the opposite sex. It is therefore, not as simple as nationality or religion.

Overall the need for cultural competence of all people involved in medical education is highlighted in this study. It is imperative that educators are culturally aware and sensitive to practices that are different in the host’s culture For example a Muslim female medical student may have concerns about shaking hands with and examining a male patient. Open dialogue on what is acceptable, and in some cases essential practice for a medical professional in patient care, is necessary in order to support students to change or adjust their individual practices. Key to improving cultural competence such as social skills and interactions is the identification of different verbal and non-verbal communication, rules, norms and practices. As noted, concern was raised around cultural competence such as understanding the rules and conventions around socially acceptable practice. For many international students the difficulty to distinguish between joking or teasing and sarcasm or being insulting acted as a barrier to social interactions across cultural groups.

Nonverbal communication norms and rules and conventions around interpersonal behaviour often present a bigger challenge to overcome than language fluency as it is often difficult to acquire this knowledge. As Masgoret and Ward note“…Such nonverbal acts often carry implicit messages that define the nature of relationships within a culture, and these messages can vary widely across cultures. In many ways, learning non-verbal forms of communication can present a bigger challenge to cultural travellers than achieving language fluency since it is often difficult to acquire the heuristic knowledge that is embedded within a culture.” [40] In addition to support programmes for academic English, the need for education around cultural competence should not be underestimated.

Another issue that arises and which is debated by Volet and colleagues [19, 41] is the categorisation of students as members of only one cultural or ethnic grouping. This regional/geographical categorisation is often the only ‘cultural’ indicator collected by academic institutions (see below). Many of the interviewees and FGD participants in our study could not be clearly classified as belonging to any one cultural, regional or national grouping; and they identified themselves as belonging to multiple groupings. Many of our students have parents or grandparents from different national or ethnic backgrounds; had lived and/or studied for long periods of their life outside their home country, e.g. many of our Canadian students were from families who had emigrated to Canada from the Middle East. Hence, self-declared ethnicities and in some cases multiple ethnicities were different to their declared nationality - or as expressed succinctly by one interviewee, ‘*I am a melting pot of cultures*’.

Globalisation has produced heterogeneous groups of international students that are different to those of two decades ago. Additionally, even when students could be classified as coming from one larger regional group, such as Middle Eastern, many agreed that there were important sub-cultures and differences within this grouping and thus the same geographical origin cannot be viewed as a homogenous cultural group. In a parallel study on the peer interviewing process for this study [25] we describe in more detail how matching peer interviewers for cultural diversity is important along a continuum of characteristics or markers as a shared background in all of the characteristics is unlikely. Students have different cultural backgrounds, even if coming from the same geographic region, and these impact on acculturation. A change in the way HEI’s gather and process data at the time of admission, reflecting students’ multiple ethnicities in the twenty-first century, would be timely. Greater disaggregation in the type of data collected at registration might assist in profiling the supports need to enhance students’ experiences and potentially identify students who may struggle in the acculturation process.

Consequently, while the interviewees and FGD participants at this HEI experience some similar stressors to those faced by international students in previous decades, as they move to a western HEI, others are new. These include the complexities and consequences of multiple ethnicities, especially where international students feel pressured or even coerced to comply with a traditional norm. Students from similar cultural backgrounds may come from differing communities within that culture; and therefore have quite different cultural values. Rienties et al. [10] discuss this phenomenon in relation to the increased mobility and internationalisation of the workforce in Europe and the greater number of students now coming from different or multi-cultural backgrounds. They argue that collecting data on parents’ nationality will often not fully capture the students’ identities. Though fees are often determined by nationality and residency, other data, such as previous overseas living, languages spoken and where educated may actually be more important in identifying students that have a potentially harder time adjusting and who may be in need of increased support services.

## Conclusion

There was considerable heterogeneity within the interviewees and FGD participants and many international students were of mixed ethnicity, multilingual and well-travelled, which can be considered a feature of globalisation of higher education in the twenty-first century. An unusual feature of this particular HEI is that 80% of all undergraduate medical students are international, placing the host country students in a minority. Hence socialising with host country (Irish) students would mean that the international students are socialising with a minority group. In such a setting, the challenges for the international student from a dominant regional grouping can be quite different, in that cultural mixing can be largely avoided by these students.

This article contributes new insights on the experiences of students coming to a western HEI in the second decade of the twenty-first century. These include the complex interrelatedness of the daily life challenges facing international students regarding the forming and the importance of intercultural relations, which is impacted by gender, the presence of alcohol, languages spoken (in addition to the host language, which in this case was the language of medical education); and the dominance of the regional groupings to which the international students belonged. The setting for this study exemplifies a “melting pot of cultures” where commonalities of cultural diversity can be seen as a counter-weight to the differences. If institutions want to remain attractive to international students then these students need to be well prepared for the experiences they will encounter in the host country setting in which they will be studying. Highlighted above are a number of suggestions to improve the experience of international and host students: such as support programmes to facilitate socialising as well as studying in the host language – in this case English, orientation on the cultures of both the host country *and* the dominant groups of international students; and, support, guidance and the provision of social activities in acceptable social venues. More disaggregated data needs to be collected when registering international students, and a more nuanced understanding of culture needs to be developed, if this information is to be used to guide support services and target interventions for students in need of assistance.

## Additional files

Additional file 1: Interview Theme sheet. Interview theme sheet for making the most of cultural diversity study. (DOCX 22 kb)

Additional file 2: Focus Group Discussion Theme sheet. Focus Group theme sheet for making the most of cultural diversity study. (DOCX 22 kb)

## Abbreviations

FGD(s): Focus Group Discussion(s)
HEI: Higher Education Institutions
ELT: Education and Life Transitions

## Footnotes

1 The term ‘international student’ is used to refer to students crossing borders for the specific purpose of studying, and the term ‘foreign student’ for non-citizens enrolled at an institution of education outside their home country, but who have not necessarily crossed a border to study. (UNESCO Institute for Statistics’ (UIS) Global Education Digest (2006), & OECD, Education at a Glance (2006)).

2 Slagging is a colloquial term in Ireland for good natured teasing.

3 The HEI introduced hours for women in the old gym subsequent to this study and has recently built a women’s’ gym
