# Supplementary material for: ‘A melting pot of cultures’ –challenges in social adaptation and interactions amongst international medical students
Source: BMC Med Educ. 2019 Mar 18;19:86. doi: 10.1186/s12909-019-1514-1 (PMC6423840; doi:10.1186/s12909-019-1514-1)
Supplement: Supplementary file 1 — Interview Theme sheet. Interview theme sheet for making the most of cultural diversity study. (DOCX 22 kb) [file 12909_2019_1514_MOESM1_ESM.docx]

**Making the most of cultural diversity**

**Interview Theme sheet**

**BEFORE INTERVIEW COMMENCES PLEASE CHECK THE FOLLOWING**:

Respondent has read the information sheet.

The information sheet is discussed with the respondent.

Confidentiality and data handling procedures have been discussed.

Consent sheet has been signed before the interview

Permission for recording the interview has been given

***Theme 1 Demographics and Background:***

Question: Respondent to tell you a little about their background.

Information sought: demographic information on: age, sex, marital status, country of upbringing, countries lived in, nationality, religion, family and importance or role family has in respondents’ life. May need prompts for this information if not volunteered by respondent.

***Theme 2 Perception of Ireland before arrival and reasons for coming to Ireland***

Question: Respondent to describe how they saw Ireland before they came and why they wanted to come to Ireland.

Information sought: perceptions of Ireland before the respondent arrived and the reasons why they (a) came to Ireland, (b) why they chose medicine, and, (c) why they chose RCSI.

For Irish students can ask how they think Ireland is portrayed and perceived abroad.

***Theme 3 Transition to Ireland and Western Europe***

Question: Respondent to describe, as far as they can remember, how it felt when they arrived in the early months and how they managed the transition to settling down to live in Ireland/Europe.

Question: Respondent to describe what was difficult for them and what helped them in this period.

Question: Respondent to describe whether this is how they expected their live to be in Ireland/Europe.

Information sought: Feelings, actions and challenges faced in living in Ireland and whether this was what they were expecting. Unpack formal support, such as orientation received, and informal support, such as fellow students, family or fellow nationals. Try to keep this specific to generally living in Ireland and not studying at RCSI.

Skip this if the student has only lived in Ireland in Dublin. If lived outside Ireland explore this; if moving from other area in Ireland to Dublin explore this.

***Theme 4 Studying Medicine***

Question: Respondent to describe how she/he found studying at Medicine.

Question: Respondent to describe what was difficult for them and what helped them in this period.

Question: Respondent to describe whether this is how they expected their medical studies would be.

Information sought: Feelings/motivation, actions and challenges faced in Medicine and whether this was what they were expecting. Again unpack formal support, such as tutorials, and informal support, such as fellow students, family or fellow nationals. Try to keep this specific to studying Medicine.

***Theme 5 Studying at RCSI***

Question: Respondent to describe how she/he found studying at RCSI.

Question: Respondent to describe what was difficult for them and what helped them in this period.

Question: Respondent to describe whether this is how they expected RCSI would be,

Information sought: Feelings, actions and challenges faced in studying at RCSI and whether this was what they were expecting. Again unpack formal support, such as orientation received, and informal support, such as fellow students, family or fellow nationals. Try to keep this specific to studying at RCSI.

***Theme 6 Recommendations***

Question: Respondent to describe what was useful when they arrived with respect to (i) living in Ireland/Western Europe, (ii) studying Medicine, and (iii) studying at RCSI.

Question: Respondent to describe what would have helped them to adjust more to (i) living in Ireland, (ii) studying Medicine, and (iii) studying at RCSI when they initially arrived.

Question: Respondent to describe what would still help them (i) to enjoy living in Ireland more and (ii) achieving better academically and enjoying life more at university.

Information sought: useful induction activities or materials, social network support, recommendations to improve induction and orientation content for students and suggestions on improving teaching and learning at RCSI.

**AFTER THE INTERVIEW PLEASE CHECK:**

Consent sheet has been signed after the interview

**Thank the respondent and ask if they would wish to add anything else.**
