# Supplementary material for: ‘A melting pot of cultures’ –challenges in social adaptation and interactions amongst international medical students
Source: BMC Med Educ. 2019 Mar 18;19:86. doi: 10.1186/s12909-019-1514-1 (PMC6423840; doi:10.1186/s12909-019-1514-1)
Supplement: Supplementary file 2 — Focus Group Discussion Theme sheet. Focus Group theme sheet for making the most of cultural diversity study. (DOCX 22 kb) [file 12909_2019_1514_MOESM2_ESM.docx]

Appendix 2

**Making the most of cultural diversity**

**FGD Theme sheet**

**BEFORE FGD COMMENCES PLEASE CHECK THE FOLLOWING**:

Respondents have read the information sheet.

The information sheet is discussed with the respondents.

Confidentiality and data handling procedures have been discussed.

Consent sheet has been signed before the FGD

Permission for recording the FGD has been given

***Theme 1 – Studying in a Multicultural Environment***

- What are your thoughts / comments on studying in a multicultural society?
- Positive / negative elements

***Theme 2 – Stereotypes***

- What are your thoughts / comments on cultural stereotypes in general?
- Do they exist?
- Are they correct?
- Do they have a positive / impact?
- Do they exist in RCSI?
- Is this a good / bad thing?

***Theme 3 – Integration***

- What are your thoughts / comments on integration in RCSI?
- Has it changed since JC1?
  - Friendship and integration - how would they define friendship?  Would they have close friends (somebody they would confide in) from other cultural groups?
  - Are their own ties within their own cultural group the same- stronger/the same/changed in anyway?  Benefits/downsides of this? Is this natural or is there pressure to remain within your own cultural grouping?

***Theme 4 – Transition to Beaumont***

- What are your thoughts / comments on issues arising from your transition to the semi-clinical environment & programme in Beaumont?

***Theme 5 – Importance of Cultural Issues in IC / JC***

- What are your thoughts / comments on the importance of cultural issues among students in the IC versus the JC?

**Topics covered should include –**

- Cultural Practices
- Alcohol
- Language
- Teaching practices
- Assessment
